# Mediterranean diet: Fighting breast cancer naturally: A review

**DOI:** 10.1097/MD.0000000000038743

**Published:** 2024-06-28

**Authors:** Yuanning Yao

**Affiliations:** a Queen Mary School, Nanchang University, Nanchang, China.

**Keywords:** adherence, breast cancer, genetic polymorphism, Mediterranean diet, prospective studies

## Abstract

The effects and mechanisms of the Mediterranean diet (MD) on the incidence, recurrence, and prevention of breast cancer (BC) have been extensively investigated since the 1990s. Recent years have witnessed significant advancements in understanding the relationship between the components of the MD and BC, particularly in terms of their role and adherence. This comprehensive review focuses on several key aspects: the influence of the adherence of MD in cohort studies conducted across different regions on BC, the effects and mechanisms of individual component or main components as well as the supplementation of vitamins, drugs, exercise, and other factors of MD on BC; the variations in the impact of the MD on premenopausal and postmenopausal women, as well as different types in BC cases; the possible mechanisms underlying the development, recurrence, and prevention of BC in relation to the MD; and the interaction effects of individual genetic polymorphisms with the MD. Based on current research findings, this review highlights the key issues and identifies future research directions in investigating the relationship between the MD and BC. Furthermore, it suggests that healthy women of various ages and BC patients should adhere to MD in order to prevent BC or improve the prognosis.

## 1. Introduction

There has been extensive research on the correlation between lifestyle and cancer for an extended period. However, due to the complexity of the research process and the multitude of variables involved, no definitive conclusion has been reached regarding which lifestyle factors can effectively prevent cancer, aid in cancer treatment, or improve cancer prognosis. Additionally, the existing studies lack the systematic scientific support which is necessary to establish firm connections. Nevertheless, after analyzing a substantial body of previous research spanning several decades, the World Cancer Society has identified certain lifestyle choices that may help prevent cancer. These include maintaining a consistent exercise routine, consuming a diet abundant in vegetables, legumes, fruits, whole grains, and breastfeeding.^[1]^ Among the different cancer-fighting lifestyles, this article examines explicitly the Mediterranean diet (MD). Significant discrepancies in cardiovascular and tumor incidence rates among countries situated along the Mediterranean coast have been conducted since the 1940s to analyze the traditional dietary patterns of these countries, commonly referred to as the MD. The exclusive exploration of the effects and mechanisms of this diet on tumor occurrence, development, and prevention has also been undertaken.^[2–4]^ In 2020, breast cancer (BC) emerged as the most frequently diagnosed form of tumor globally, surpassing all other types. Additionally, it remains the foremost women’s death affected by cancer.^[5]^ More and more evidence indicates that BC is closely related to lifestyle.^[6]^

Studying the correlation between lifestyle and BC is paramount in enhancing global health and alleviating BC treatment’s social and economic strains. Nonetheless, the existing research surrounding the influence of the MD on BC yields conflicting results. Moreover, numerous theories exist regarding the mechanisms underlying the impact of MD on BC, but a comprehensive summary of the potential associations between the 2 still needs to be provided. Thus, this review aims to present the principal research findings concerning the effects and mechanisms of the MD on the impact of BC while concurrently identifying mutually comparable connections between them.

## 2. Introduction to MD and its relationship with cancer

### 2.1. Introduction of MD

In 1957, Keys et al proposed that the variations in dietary fat intake and subsequent increases in serum cholesterol levels were responsible for the varying incidences of coronary heart disease in different global regions.^[7]^ In the 1960s, research on the relationship between the traditional dietary patterns of olive-growing areas of the Mediterranean (such as Spain et al) and health drew attention. These nutritional patterns were later recognized as MD and gained lots of attention. In 1993, the World Health Organization et al organization collaboratively introduced the MD pyramid model. Additionally, in 1997, Trichopoulou et al provided a comprehensive overview of the Mediterranean region’s traditional dietary pattern, culture, history, and lifestyle,^[8]^ In 2001, Noah et al interviewed 102 individuals from 18 Mediterranean countries in Sydney to investigate dietary habits. The study revealed similarities and significant differences in nutritional practices among these nations. It is important to note that there is no universally accepted standard MD pattern.^[9]^

Currently, there are multiple definitions of the MD, and a consensus has yet to be reached regarding its precise definition. Nevertheless, many publications share a common description of the MD, highlighting similar key components. These definitions emphasize the importance of consuming substantial quantities of extra virgin olive oil, grains, fruits, and nuts. It is also been stressed that fish meats other than red meat while limiting the intake of sugars, etc.^[10]^ This dietary pattern stands in stark contrast to the Western diet, which primarily consists of processed foods and red meat.

### 2.2. Introduction of the relationship between MD and cancer

Since the 1980s, there has been considerable attention on how the MD relates to cardiovascular disease and cancer. Multiple laboratories have been conducting large-scale cohort studies on MD and diseases. For examples, in 2003, Trichopoulou et al conducted a prospective study (NEJM) that evaluated adherence (MD score: 0 to 9) in 22,043 adults in Greece. The results showed that greater MD adherence was associated with a lower overall mortality rate.^[11]^ In addition to cardiovascular diseases, the close correlation between over 20 human tumors and MD has received widespread attention and research results support. For example, in 2004, Gallus et al analyzed the 12,000 cancer cases comprising 20 different types of cancers, comparing them with a control group of 10,000 individuals in northern Italy. The study covered a period from 1983 to 2001 and yielded significant findings regarding certain dietary components to mitigate tumor risks. The research revealed that an augmented intake of vegetables and fruits reduced the risk of epithelial tumor. Similarly, whole-grain foods were found to diminish the likelihood of digestive tract cancer. Furthermore, a fiber-rich diet was observed to protect colorectal tumor. Lastly, it is important to note that increasing vegetables and reducing meat intake would decrease the risk of tumor development. These dietary recommendations align with the composition of the MD, which is renowned for its favorable effects in reducing cancer risks^[12]^; in 2015, Schwingshackl et al studied a total of 1784,404 participants (meta-analysis), They revealed the association between high adherence to the MD and a reduced risk of various types of tumor mortality: high relationships: colorectal, breast, stomach, prostate, pancreatic, liver, head and neck tumor, etc. No significant relationships: ovarian, endometrial, bladder tumor, etc.^[13]^ Meanwhile, in 2017, they further studied a total of 2,130,753 participants (randomized trials, cohort studies, meta-analysis) and reached similar conclusions, furthermore, they separately analyzed the risk reduction of breast cancer.^[14]^ According to a scientific review published in 2023, which examined articles in the PubMed database from January 1, 2000, to June 12, 2023, it was found that the MD is associated with a reduced risk or shows no significant impact on different types of cancer.^[15]^ Although studies have reported on the relationship between MD and multiple tumor cohorts, there are currently limited comparisons regarding the differential impact of MD on various tumors. Future research will focus on investigating the variations in the effects and mechanisms of MD on different types of tumors. The objective of this review is to specifically examine the research progress concerning the pathogenesis, recurrence, prevention, and potential mechanisms of the MD in relation to BC.

## 3. The impact of the MD on the incidence, recurrence, and prevention of BC

Various factors contribute to the occurrence and progression of BC. These factors include genetic predisposition, such as a family history of the disease, and mutations in genes like breast cancer susceptibility gene 1/2 (BRCA1/2). Other risk factors include age, age of first onset of menstruation, delayed pregnancy, dense breast tissue, smoking and alcohol consumption, lack of physical activity, an unhealthy diet, obesity, hormone replacement therapy, the use of oral contraceptives, and so on. In recent years, there has been growing interest in the MD and its potential impact on reducing the occurrence, recurrence, and prevention of BC.

Genetic predisposition (such as a family history of the disease, and mutations of breast cancer susceptibility gene 1/2), hormone replacement therapy, age and age of first onset of menstruation, delayed pregnancy, dense breast tissue, smoking, an unhealthy diet, obesity, and so on would contribute to the occurrence and progression of BC. Currently, MD and its potential impact on reducing the occurrence, recurrence, and prevention of BC have received widespread attention.

### 3.1. Main research methods on the impact of MD on BC

The study examining the impact of MD on BC is inherently intricate, and there are notable disparities in research methodologies and diverse populations across various regions. In general, the predominant research model employed is:

1. Prospective and retrospective studies are used to examine the variations in regions, premenopausal and postmenopausal states, age, and other factors in the study population.
2. Designing diverse diet plans based on the traditional MD model.
3. Multiple survey questionnaires and standards are used to estimate the compliance of participants.
4. Developing different diet plans for various researchers to conduct comparative studies.
5. Selecting and evaluating different monitoring indicators before and after the dietary intervention.
6. Designing and choosing the types, duration, and endpoints of the intervention in the experiment.
7. Selecting appropriate statistical methods based on different research objectives and subjects. The primary statistical indicators encompass hazard ratios, odds ratios, multivariable-adjusted relative risk, and reduced rank regression, among others.
8. How to select the main statistical models.
9. Offering logical dietary recommendations based on the obtained outcomes.

In the investigation of the correlation between the MD and BC, how to judge adherence to the MD is the first challenging step. Currently, there is a lack of standard methods and assessment indicators to objectively measure adherence to this diet. Additionally, the design and implementation of assessing adherence to the MD are highly intricate. In 1995, Trichopoulou et al created a predesigned MD adherence score based on 8 characteristics of the main diet in the Mediterranean region. This score had been used to evaluate the link between adherence and overall mortality rates.^[16]^ Various subsequent researchers have developed their criteria for assessing adherence to the MD in their studies. These researchers design questionnaires to evaluate different aspects, employing appropriate monitoring indicators and statistical methods to measure adherence.^[17]^ A study on 10 different MD indices reveals variations in the evaluation outcomes, but overall the evaluation results are relatively reliable.^[18]^ The adherence score for the MD is commonly assessed on a scale of 0 to 10 by researchers to facilitate investigation. Subsequently, statistical analysis is conducted to compare high adherence, moderate adherence, and low adherence to the diet.

### 3.2. The benefits of the MD on breast tumor cohorts in different regions

Several cohort studies have been reported in various regions to estimate the impact of the MD on BC due to variations in dietary habits and adherence to the diet across different regions. Overall, the author of this article suggests that following an MD can decrease the incidence and recurrence of BC and enhance prognosis. Nevertheless, the effects of the MD on BC can vary depending on geographical location.

#### 3.2.1. The MD overall reduce the incidence and recurrence rate of BC

In 2012, Demetriou et al studied 935 BC patients and 817 healthy controls, the results showed no significant association between the risk of BC and the adherence score to the MD by the logistic regression model. However, they did observe that a higher consumption of olive oil, vegetables and fish was independently linked to a reduced risk of BC. It suggests that following the special MD may have a positive impact on the risk of BC.^[19]^ In 2018, Turati et al studied the correlation of BD and BC, they encompassed 3392 healthy controls and 3034 BC patients from Italy and Switzerland. The findings revealed that adhering to the MD would decrease the risk of BC in both postmenopausal and premenopausal women.^[20]^ Krusinska et al developed a scoring system known as the Polish-adapted Mediterranean diet to estimate adherence to the MD in Poland. They aimed to explore the relationship between the Polish a-MED score and the risk of lung or breast tumor in Polish adults. The research encompassed 560 participants from the northeastern region of Poland, comprising 280 males and 280 females, with 140 female BC patients and 140 male lung cancer patients. The findings revealed that individuals with an average (score 3 to 5) or high adherence (score 6 to 8) to the Polish a-MED had a 51% lower risk of lung or breast tumor compared to the low adherence group (score 0 to 2). These results suggest that adopting the Polish a-MED is a preventive measure against BC who reside in the countries of the non-Mediterranean region.^[21]^ In 2020, Di Maso et al conducted a study in northern Italy involving 1453 women diagnosed with BC between 1991 and 1994 (with a 15-year follow-up). The findings revealed that women who adhered closely to the MD (n = 500, 34.4%) had a more favorable prognosis compared to those who had limited adherence to the diet (n = 332, 22.8%). The high adherence group’s survival rate (15 years) was 63.1%, but the low adherence group was 53.6%. These results suggest before their BC diagnosis who followed the MD may have a higher likelihood of achieving a positive prognosis.^[22]^ In 2021, La Torre et al evaluated the synergistic impact of noncompliance with the MD on the incidence of BC. The study consisted of 94 BC patients and 88 healthy controls. The findings of the study revealed that an MD score above 6 was linked to a reduced likelihood of developing BC. Additionally, the study demonstrated an additive and synergistic effect between noncompliance with the MD and multiple risk factors for BC.^[23]^ Overall, the results of MD and BC are generally consistent among different populations and laboratories in different regions, but there are also some contradictory results, which may be due to differences in research methods and cultures, resulting in inconsistent adherence with MD.

#### 3.2.2. Certain studies indicate a lack of robust correlation between the MD and BC

At the same time, in research on the connections between MD and BC, some study results suggest that there is no direct correlation between MD with BC. In 2013, Couto et al investigated the relationship between MD and BC, they recruited a total of 49,258 women aged 30 to 49 in Sweden during 1991 to 1992. Among the participants, 1278 cases of BC were diagnosed. Results indicated that no significant relationship between MD and the risk of BC or specific characteristics of breast tumors.^[24]^ In 2014, Pot et al conducted a study using MD scores, they examined the relationship between 610 BC patients and 1891 healthy (cohort studies in the UK). The study found no significant relationship between MD scores and the risk of BC. However, the analysis revealed that high alcohol intake correlated with an increased BC risk BC.^[25]^

### 3.3. Effects of the MD alone or in combination with other components interventions on BC

Several research laboratories have conducted studies on the primary MD components to affect BC. Moreover, they have compared the MD with other dietary patterns and investigated the effects of including exercise, vitamins, medications, and alternative methods in the MD on BC.

#### 3.3.1. The main MD constituent effects on BC

In terms of the inherent components of the MD, in 2014, Mourouti et al evaluated the relationship between MD and BC. They assessed the MD scores of 250 BC patients and 250 healthy controls. The results suggested that the most important components and beneficial effects of the MD in preventing BC may include vegetables, fruits, alcohol, and unrefined grains.^[26]^ In 2019, a comprehensive review by Shaikh et al examined the components of MD effects on BC. including polyphenols (organosulfur compounds, epigallocatechin gallate, quercetin resveratrol, apigenin, kaempferol), fatty acids, trace nutrients (selenium, zinc), etc, with a particular focus on diet and individualization. The authors also emphasized the significance of considering individual genetic responses to diet, which will be further elucidated in the mechanisms section of this article. Furthermore, this study highlighted the advantageous role of nutritional genomics in BC prognosis.^[27]^

The MD is predominantly studied for its primary constituent, polyphenols. Polyphenols in the diet are primarily derived from flavonoids present in tea, phenolic acids in olive oil, isoflavones, grapes, and chocolate in soy products. Numerous laboratory studies have examined and documented the presence of phenolic acids within the MD. In 2016, Braakhuis et al reviewed the dietary pattern rich in polyphenols, completed clinical trials aimed at preventing BC recurrence, mechanistic data, and innovative drug delivery methods for polyphenols. The primary mechanisms of polyphenols were summarized as follows: reducing inflammation and likelihood of cancer recurrence through antioxidant effects, increasing histone deacetylase activity, inhibiting cancer cell proliferation, and preventing pro-inflammatory cytokines, kinases, and transcription factors. The author recommends that individuals with BC consume a natural diet rich in flavonoid polyphenols (tea, onions, cauliflower, fruits, etc). MD is suggested as the most promising dietary pattern for BC patients.^[28]^

How the phenolic acids affect BC, in 2020, Romanos-Nanclares et al found a negative correlation between the intake of hydroxycinnamic acid and BC (10,812 participants). The results showed that chlorogenic acid (3-, 4-, and 5-caffeoylquinic acid) exhibited the strongest negative correlation.^[29]^ More laboratories are needed to conduct joint research on the many unclear monomeric components in MD.

#### 3.3.2. The impact of supplementing specific ingredients in the MD on BC

In 2015, Toledo et al examined 2 interventions (extra-virgin olive oil, mixed nuts) on BC occurrence. A total of 4282 participants participated in the study, and their progress was monitored for 4.8 years, 35 cases of BC were diagnosed. The findings showed that incorporating extra-virgin olive oil into MD demonstrated significant advantages in preventing primary BC.^[30]^ In 2013, Voevodina et al studied vitamin and mineral supplements, and alcohol consumption on breast density. The study included a total of 424 participants, encompassing both pre- and postmenopausal individuals aged 21 to 84 years. The results revealed a potential protective effect against BC, as individuals who followed an MD and used multiple vitamin and mineral supplements exhibited decreased breast density. In contrast, high alcohol intake increased breast density.^[31]^ In 2020, Cho et al studied MD alone or supplement with canagliflozin/afatinib, they enrolled a total of 44 BC survivors and demonstrated that both intervention groups improved metabolic parameters, resulted in weight loss, and enhanced quality of life.^[32]^ One of the main challenges in conducting research is how to supplement synergistic components in MD.

#### 3.3.3. Comparing the effects of incorporating the MD with other approaches on BC

Multiple laboratory studies on MD and BC by increasing physical exercise, designing comparative diets, and incorporating special monitoring indicators, among other methods. These studies have obtained multiple beneficial outcomes. In 2012, Villarini et al studied the effectiveness of the MD and moderate physical activity. The study included 1208 volunteers (an intensified diet, and exercise intervention group). The intervention group received cooking classes, had structured meals, and participated in exercise courses. Both the control group and intervention group are followed for 5 to 7 years. The study findings showed comparable rates of negative tumors (22%) and axillary lymph node metastasis (42%) between the 2 groups. Furthermore, reproductive variables, smoking habits, blood pressure, body measurements, as well as hormone and metabolic parameters, were similar in both groups. The intervention method showed the potential to reduce BC recurrence.^[33]^ In 2017, Kiechle et al recruited participants who tested positive for BRCA1/2. The intervention group underwent a structured and personalized endurance training program for 3 months receiving MD. The findings indicated that the lifestyle intervention potentially had a positive effect on body mass index, dietary habits, and stress levels. However, to establish the reliability of these findings, further large-scale randomized trials are necessary.^[34]^ There are currently too many difficulties in conducting queue comparison studies using different methods.

### 3.4. The differential effects of MD adherence on different populations and different pathological types of BC

There are currently multiple studies examining the MD and BC in various populations, as well as the risk of different pathological subtypes of BC. However, the findings from these studies are inconclusive. This article provides a comprehensive overview of the impact of MD on the occurrence of BC in different pathological subtypes of BC.

#### 3.4.1. The varying influences of adhering to MD on the susceptibility to BC in pre- and post-menopausal women

Overall, based on existing literature, adherence to the MD is more strongly associated with BC risk in postmenopausal women. In 2010, Trichopoulou et al studied 14,807 female participants (240 cases of BC were diagnosed). The results showed traditional MD might be linked to a lower risk of BC only in postmenopausal women.^[35]^ However, in 2011, Cade et al studied a total of 33,731 female participants, including 828 diagnosed BC patients, about MD and BC no clear relationship was observed among postmenopausal women.^[36]^ In 2012, Buckland et al studied 62,284 postmenopausal women (1256 cases of primary invasive BC were identified). The study did not find a relationship between olive oil consumption and the risk of ER+ or PR+ tumors. But olive oil consumption and hormone receptor-negative tumors showed a significant inverse association.^[37]^ In 2015, Farsinejad Marj et al (5 cohorts and 3 case controls) found that adhering to an MD diet may have a protective effect on both postmenopausal and premenopausal women.^[38]^ In 2017, Castell ó et al studied 1181 female breast cancer patients and 1682 healthy controls in 10 provinces of Spain. The results suggest that high adherence to the Western diet model seems to increase the risk of premenopausal women suffering from breast cancer, but the MD model seems to have a protective effect, especially in postmenopausal women.^[39]^ In 2018, Coughlin et al reviewed the relationship between the expression of estrogen receptor (ER), progesterone receptor (PR), human epidermal growth factor receptor 2 and MD adherence (6 cohort studies, 1 case-control study, selected from 154 articles from 1985 to 2017), and found that the MD pattern was negatively correlated with the risk of postmenopausal women suffering from breast cancer.^[40]^ In 2020, Laudisio et al put forward in their review that there is not enough scientific evidence to support that premenopausal women’s adherence to the MD pattern is related to the reduced risk of breast cancer or specific characteristics of breast cancer.^[41]^ Overall, postmenopausal women may benefit more from MD, but further research is needed to understand the mechanisms underlying the differences compared to premenopausal women.

#### 3.4.2. The impact of MD adherence on the differential risk of BC by molecular subtypes

The classification method commonly used for BC in clinical practice is based on the molecular receptor type, which includes HER2, ER, and PR. These molecular receptors play an important role in guiding treatment decisions and prognostic evaluations for BC patients, offering more individualized and precise treatment plans. While the benefits of adhering to the MD have been proposed for ER-negative. ER and PR negative, and triple-negative BC (triple-negative breast cancer [TNBC], PR-, ER-, HER2-). There is a lack of evidence indicating that the MD has an impact on other types of BC such as ER positive. In 2012, Buckland et al studied 335,062 female participants recruited in 10 European countries from 1992 to 2000, and diagnosed 9009 cases of postmenopausal breast cancer, 1216 cases of premenopausal breast cancer, 5862 cases of ER‐ and PR+, or ER+PR‐, and 1018 cases of ER‐ and PR‐. The results suggest that MD without alcohol is related to reducing the risk of breast cancer, and is stronger in ER‐ and PR‐.^[42]^ In 2014, Castell ó et al studied 1017 cases of sporadic breast cancer in Spain and 1017 healthy controls and found that the MD mode had a stronger protective effect on triple-negative tumors.^[43]^ In 2017, Van et al studied the diet and lifestyle information of 62,573 Dutch women aged 55 to 69 years (since 1986), and found that MD compliance was significantly negatively correlated with ER-negative breast cancer risk, and weakly negatively correlated with ER-positive or breast cancer total risk.^[44]^ In 2018, Coughlin et al also found that the MD pattern was negatively correlated with the risk of ER breast cancer.^[40]^ How to conduct mechanism research on the one-to-one correspondence between MD and ER, PR, and HER2 is an important research direction.

## 4. The potential mechanisms underlying the effects of the MD on BC

The impact of the MD on BC is currently being investigated by researchers to understand the underlying mechanisms. It is believed that while individual ingredients may play a role, the benefits of this diet in relation to BC are primarily attributed to the synergistic effects of all its components. These researchers are exploring the impact of the MD on BC through various avenues, including the examination of BC cells, animal models, and the consideration of individual genetic backgrounds.

### 4.1. The mechanisms of action of the MD in BC cell lines and experimental BC models

In 2006, Menendez et al made a significant discovery regarding the effects of the MD on BC. Specifically, they found that α-linolenic acid which is present in the “fat profile” of MD and is part of the “anti-HER2 cocktail,” effectively suppressed the HER2 transcriptional level. This groundbreaking research was conducted on HER2-positive BC cell lines, namely BT-474 and SKBr-3.^[45]^ The author also discovered that in tumor-derived cell lines SK-Br3, SKOV3, and NCI-N87 (HER2 positive), monounsaturated fatty acid oleic acid-induced the transcriptional repression of HER2 through the action of the Polyomavirus enhancer activator. This may represent a new mechanism of the MD’s anticancer properties.^[46]^ In 2011, Escrich et al analyzed the 16 experimental series using animal models, the results revealed that a diet enriched with extra virgin olive oil exerted a negative regulatory effect on experimental BC, and induced various molecular changes within tumors, such as reduced DNA damage, etc.^[47]^ In 2020, Donovan et al studied the impact of olive oil, fish oil, etc in the context of TNBC models between 2015 and 2019. The study offered an overview of the different constituents of MD oil that target established aberrant signalling pathways in TNBC, such as phosphoinositide 3-kinase-protein kinase B-mammalian target of rapamycin, nuclear factor-kappa B-cyclooxygenase-2, and wingless-Int-1-β-catenin.^[48]^

In 2020, Benot Dominguez et al studied the effect of olive leaf extract on TNBC and ovarian cancer cells. In the experimental study of tumor cell lines (HCEpiC, OVCAR-3, MDA MB-231), they found that olive leaf extract can induce cell cycle arrest and induce cell death through poly ADP ribose polymerase and cysteinyl aspartate specific protein 9.^[49]^ In 2020, Messeha et al used TNBC cell lines MDA-MB-468 and MDA-MB-231 and found that oleuropein can effectively inhibit cell growth, accompanied by S-phase cell cycle arrest mediated apoptosis.^[50]^

### 4.2. The possible interaction between the MD and individual genetic polymorphisms in BC

In 2012, Vanden Berghe et al the epigenetic effects of dietary polyphenols (soy, curcumin, resveratrol, catechins, and gingerols) in the prevention of cancer. Their findings suggested that polyphenols present in vegetables and fruits can reduce the risk of cancer. Various factors that can influence these effects, such as the timing and dosage of dietary exposure, as well as confounding factors like smoking and alcohol consumption.^[51]^ In 2015, Kakkoura et al studied the relationship between MD and BC. It specifically focused on the impact of 2 enzymes related to the one-carbon metabolism pathway, one is methylenetetrahydrofolate reductase (MTHFR), and the other one is methionine synthase (MTR). Researchers examined if the single nucleotide polymorphism (SNP) genes of these 2 enzymes (rs1801133 and rs1801131, MTR:rs1805087) related to the BC and MD. By conducting a principal component analysis on the diet patterns, the researchers found that these SNPs in MTR and MTHFR might act as modifiers in MD and BC.^[52]^ In 2016, Kakkoura et al studied whether manganese-containing superoxide dismutase (MnSOD) and catalase (CAT) are related to the BC risk and the MD. After examining the SNPs of these 2 enzymes (MnSOD: rs4880; Val16Ala. CAT: 262C>T, rs1001179). In BC cases from Cyprus, it was observed that the presence of wild-type MnSOD or CAT SNPs may enhance the antioxidant effect of the MD on BC.^[53]^ In 2017, Kakkoura et al studied the potential impact of glutathione S-transferase pi-1 and arylamineN-acetyltransferase-2 SNPs. Their findings indicated that a high adherence to the MD can reduce the risk of BC by anticarcinogenic effects.^[54]^ Meanwhile, Kakkoura et al aimed to estimate adherence to the MD, specifically by vegetable, fruit, legume, and fish consumption, among Greek-Cypriot women categorized into groups with the lowest or highest levels of BC. Principal component analysis was employed for this evaluation. The researchers made an interesting discovery that the interaction between serum lutein mononucleotide and 5-methyltetrahydrofolate levels is impacted by MTHFR (rs1801133) polymorphism and GSTM1 deletion.^[55]^ In 2018, Pasanisi et al conducted a 6-month study involving 213 carriers of BRCA gene mutations to estimate how the influence of MD on serum levels of insulin resistance and insulin-like growth factors related to the BRCA gene mutations. The intervention group took part in a 6-day lifestyle intervention program, which included cooking classes, followed by lunch, a 45-minute walk, and nutrition meetings. The researchers discovered a potential influence of Insulin-like Growth Factor-1 levels on the penetrance of BRCA mutations in women from the intervention group.^[56]^ In 2019, Shaikh et al conducted a comprehensive review focusing on “personalized” research between BC and MD. The review emphasized the role of various components found in the MD, as well as trace nutrients like zinc and selenium, and their impact on BC. Additionally, the authors provided an overview of the genetic background and nutrition influences the BC.^[27]^ In 2021, Cao et al studied how adherence to the MD and SNPs of one-carbon metabolism impact BC risk. They conducted the study on a population of Chinese women, including 818 BC patients and 935 healthy controls. The researchers performed SNP testing on 8 genes and found it reduced the risk of postmenopausal women developing BC. The mechanism behind this reduction in risk may be associated with mitigating the genetic susceptibility related to one-carbon metabolism in postmenopausal women.^[57]^

### 4.3. Additional potential mechanisms through which the MD impacts the development of BC

In 2006, Carruba et al studied the impact of the MD on endogenous estrogen levels. They randomly assigned volunteer women to 2 groups (intervention group, n = 58. control group, n = 57). The results suggest that establishing BC dietary prevention measures from an estrogen perspective could be supported.^[58]^ In 2008, Tseng et al aimed to investigate the relationship of MD and breast density. In 1286 participants, they did not find a significant association between the MD and the percentage of breast density. However, it suggested that the MD may have a protective effect primarily in the presence of carcinogenic compounds.^[59]^ In 2018, Shively et al utilized nonhuman primate models, revealing that the consumption of either a Western diet or an MD would influence the breast metabolite profile and microbiome. This finding suggests the potential establishment of an alternative mechanism for preventing BC.^[60]^ Meanwhile, in 2019, Newman et al presented an overview of how Mediterranean and Western dietary patterns can influence microbiota-mediated signalling, thereby affecting the risk of BC. The study revealed that the MD can cause substantial alterations in the microbial composition of breast tissue, suggesting a potential anticancer effect. This finding highlights the significance of diet in shaping the specific microbiota of the breast and its potential impact on cancer prevention.^[61]^ In 2020, Kwon et al conducted a study aimed at analyzing the expression levels of 798 micro RNAs (miRNAs) in participants who followed the MD for a duration of 8 weeks. Following this 8-week period, significant differences were observed in the expression levels of 42 extracellular vesicle miRNAs, with 36 of them being upregulated and 6 being downregulated. These findings suggest that the differentially expressed extracellular vesicle miRNAs, which may have been influenced by the MD may potentially affect the mechanisms associated with metabolic risk factors among overweight BC survivors.^[62]^ In 2020, Szamreta et al studied MD during ages 9 to 10 and breast development (menarche) and the onset of first menstruation. The study found that the MD may be associated with a later onset of menarche and first menstruation at a higher age.^[63]^ Multiple laboratories have reported on the specific molecular mechanisms of MD’s effect on BC in queue studies and cell model studies with different genetic backgrounds, but more research is needed.

## 5. Conclusion

Recent studies have made significant progress in exploring the relationship between the MD and BC. Several cohort studies have provided valuable insights into its benefits, molecular mechanisms, and dietary recommendations. However, there are still some key issues and future prospects to consider. Firstly, it is important to expand the MD and BC cohort studies to include more regions. Secondly, in a general description, the MD is characterized by “olive oil, grains, fruits, and nuts” as its primary research components, and “fish, poultry, and moderate wine consumption” as supplementary research components.^[7–10]^ However, diverse criteria have been applied in practical studies on the Mediterranean diet, leading to challenges in comparing the outcomes. It is essential to promote a specific criteria for the components to enhance comparability across various studies. Thirdly, in the future, it is required to investigate the molecular mechanisms and synergistic actions of individual components within the MD. Additionally, as BC incidence is rapidly increasing in developing countries, exploring the preventive effects of MD through interventions becomes crucial. Lastly, investigating the impact of personalized genes and microbial factors on the anticancer effects of MD is an important research direction. In terms of overall recommendations regarding BC occurrence, recurrence, and prevention in relation to the MD: Firstly, postmenopausal women who adhere to the MD can potentially reduce their risk of BC. Secondly, postmenopausal BC patients can benefit from incorporating the MD into their eating habits. Thirdly, adhering to the MD may potentially reduce the risk of recurrence in ER- or TNBC patients. Fourthly, it needs to compare the Western diet and MD impact of the BC incidence in premenopausal women, and more evidence and research are needed to determine the specific benefits of the MD for premenopausal women. Lastly, the mechanisms of action of the MD and its interaction with susceptibility genes and polymorphisms related to one-carbon metabolism are closely intertwined, suggesting individual variations in the benefits derived from the MD. The MD pattern itself may not be a direct cause of reduced cancer risk; rather, it is likely the higher intake of legumes, fish, and vegetables that plays a significant role.

## Author contributions

**Conceptualization:** Yuanning Yao.

**Investigation:** Yuanning Yao.

**Methodology:** Yuanning Yao.

**Resources:** Yuanning Yao.

**Validation:** Yuanning Yao.

**Writing – original draft:** Yuanning Yao.

**Writing – review & editing:** Yuanning Yao.
